# Effect of *Lactiplantibacillus plantarum* LPJZ-658 on caecum microbiota and serum metabolomics of Luhua broiler

**DOI:** 10.3389/fmicb.2025.1622009

**Published:** 2025-08-20

**Authors:** Mengjiao Li, Zhongyuan Liu, Chen Chen, Ziqi Liu, Liming Liu

**Affiliations:** ^1^Department of Preventive Veterinary Medicine, College of Veterinary Medicine, Northwest A&F University, Yangling, China; ^2^College of Animal Science and Technology, Jilin Agricultural Science and Technology University, Jilin, China

**Keywords:** *Lactiplantibacillus plantarum* LPJZ-658, Luhua broiler, 16S rRNA, cecal microbiota, serum metabolomics

## Abstract

**Introduction:**

The purpose of this study was to investigate the effects of dietary supplementation of *Lactiplantibacillus plantarum* LPJZ-658 on body weight and serum indexes of Luhua broiler, and to explore the relevant mechanism of probiotic function of LPJZ-658 based on intestinal microbiota and serum metabolomics.

**Methods:**

One hundred one-day-old Luhua broiler were randomly divided into the control group (CON) and LPJZ-658 treatment group (LPJZ-658). The CON group was fed a basal diet, and the LPJZ-658 group was fed the basal diet supplemented with 2 × 10^9^ cfu/kg of LPJZ-658. The study lasted for 28 days. At the end of the experiment, serum and caecum samples were collected for analysis.

**Results:**

In the LPJZ-658 group, the serum IgA level, and activity of SOD were significantly higher, concentration of MDA was markedly lower than in the CON group. Caecum microbiota showed that LPJZ-658 could dramatically change the composition of cecum flora. It’s mainly by increasing the level of Lactobacillus, Lachnoclostridium, and Parasutterella, and reducing the level of Clostridia_UCG-014, Faecalibacterium, Blautia, Eubacterium_coprostanoligenes_group, Anaerofilum and Shuttle. In addition, serum non-targeted metabolomics results showed that there were 49 serum differential metabolites between the two groups, and the main metabolic pathways affected by LPJZ-658 included phenylalanine, tyrosine and tryptophan biosynthesis, arachidonic acid metabolism, and tyrosine metabolism.

**Discussion:**

In summary, LPJZ-658 can improve the serum immune performance and antioxidant capacity of Luhua broiler by regulating the composition of caecum microbiota and serum metabolome, thus improving the health status of Luhua broiler and culture efficiency.

## 1 Introduction

Poultry breeding has been greatly intensified in terms of scale and feeding mode. The large-scale breeding industry may alleviate the contradiction between supply and demand in the agricultural market (He, 2020). Antibiotics are widely added to feed to prevent and treat infectious diseases and to improve the growth performance of livestock. However, the use of large doses of antibiotics in animals has raised food safety problems and environmental pollution, and led to increased bacterial resistance (Ramos et al., 2013). The comprehensive ban on antibiotic growth promoters (AGP), has led to increased research on AGP alternative supplements (Alloui et al., 2013).

Many products, including probiotics, have been approved as alternatives to antibiotics in animal feed. Probiotics have received more attention due to their antagonistic effect on a variety of microorganisms and significant growth promotion effect, and have been considered as an important and promising alternative to antibiotic additives (Wang and Kim, 2021). Studies have shown that probiotics are important in modulating immunological response, inhibiting intestinal inflammation, and preventing tissue damage (Ferreira et al., 2011). In some animal studies, different probiotics have shown facilitating effects, including improving growth performance, immune function, and antioxidant capacity (Koenen et al., 2004; Cui et al., 2019; Song B. et al., 2022). In recent years, *Lactiplantibacillus plantarum*, one of the probiotics commonly used in the fermentation process, has been reported to promote the growth and development of poultry, and it is widely used in poultry farming (Liu et al., 2023b; Wang et al., 2023; Yuan et al., 2023).

Accumulated findings established the close relationship between the improvement of gut microbiota by *L. plantarum* and poultry growth performance (Johnson Timothy et al., 2018; Al-Khalaifa et al., 2019; Yang et al., 2024). The composition of intestinal microbiota is important for maintaining gastrointestinal homeostasis and host health. In mammals, there is growing evidence that interference with early intestinal microbial colonization can have long-lasting beneficial or detrimental effects on individual health (Benis et al., 2015; Korpela and de Vos, 2018). Notably, unlike mammals, poultry embryonic development occurs in the eggs removed from the maternal environment, so the establishment of intestinal microflora is relatively lacking guidance from the maternal microflora and is mainly influenced by the postnatal environment and diet after birth. However, there are fewer experimental studies on the effect of *L. plantarum* on intestinal microbiota from birth in broiler.

In our previous study, *L. plantarum* LPJZ-658 significantly improved growth performance and egg quality in late-laying hens. Moreover, LPJZ-658 supplementation significantly increased growth production, improved meat quality and intestinal status, and modulated the intestinal microbiota in the broiler (Liu et al., 2023b). In this context, we evaluate the role of LPJZ-658 as a feed additive on growth, microflora modulation, and serum metabolism in Luhua broilers. The present study aimed to investigate the effect of the application of LPJZ-658 from newborn until 28 days feed on Luhua broiler body weight, immune function, antioxidant capacity, gut microbiota, and serum differential metabolites by the combinatorial approach of sequencing 16S rRNA analyses and non-targeted metabolomics. To provide a theoretical basis for the application of LPJZ-658 in chicken production.

## 2 Materials and methods

### 2.1 Probiotic strain and ethical approval

*Lactiplantibacillus plantarum* LPJZ-658 was originally isolated and preserved in our laboratory (Deng et al., 2023). LPJZ-658 was added to the basal diet as a freeze-dried powder, which contained 2 × 10^12^ cfu/kg. The powder was added to the basal diet at 1 g/kg to provide 2 × 10^9^ cfu/kg of diet. The bacteria and basal diet were mixed homogeneously every day. The study was approved by the Animal Care and Use Ethics Committee of Jilin Agricultural Science and Technology University.

### 2.2 Experimental design

A total of 100 1-day-old healthy Luhua broilers with similar initial weights were randomly assigned to two treatment groups: CON group was treated with basal diet, while LPJZ-658 group was supplemented with LPJZ-658 to the basal diet. Each group has five replicates of 10 Luhua broilers. The study was carried out for 28 days. The broilers in each group were weighted on days 1, 7, 14, 21, and 28.

For the study, a 24-h constant-lighting program was used and broiler were allowed free access to diets and water. The room temperature in the first week was set at 35°C and then gradually decreased to 25°C until the end of the experiments. All diets were antibiotic-free and were formulated to meet the nutrient requirements for Luhua broiler (Supplementary Table S1).

### 2.3 Sample collection and measurements of biochemical parameters in serum

Feed was removed from all pens 12 h before slaughter. On day 28, one Luhua broiler was randomly selected, weighted, euthanized, and sampled from each replicate of the CON group and LPJZ-658 group. Samples of the thymus and spleen were removed and weighed. Blood samples were collected at room temperature for 30 min and then centrifuged at 3000 r/min and 4°C for 10 min. After centrifugation, the superserum was frozen at −20°C. The concentration of IgA, IgG, IgM, MDA (malondialdehyde), T-AOC (total antioxidant capacity), and the activity of SOD (superoxide dismutase), GSH-PX (glutathione peroxidase) were measured using commercially available chicken Enzyme-Linked Immunosorbent Assay Kit (Nanjing Jiancheng Institute of Bioengineering, Nanjing, China).

### 2.4 Cecum microbiota analysis

The cecal contents of Luhua broiler were collected and stored at −80°C. Samples were stored and sent to Novogene Co. (Beijing, China) for 16S rRNA sequencing. The V3 and V4 hypervariable regions of 16S rRNA were selected to generate amplicon and then analyzed. Analysis of bacteria community data was conducted using the NovaMagic platform (https://magic.novogene.com/; accessed on 14 May 2024).

### 2.5 Serum metabolomics analysis

Two groups of Luhua broiler serum samples (*n* = 10) were selected for metabolomics analysis (Liu et al., 2023a). Each serum sample was taken 50 μL and placed in a 1.5 mL EP tube, added 150 μL methanol, swirled for 30 s, and placed in a refrigerator at −20°C for 1 h. The mixed solution was then centrifuged at 4°C for 12,000 r/min for 15 min, supernatant was taken and lyophilized using a freeze dryer. Add 200 μL of methanol/water (1:1, V/V) solution for re-dissolution, centrifuge at 4°C for 15,000 r/min for 15 min, and take the supernatant into the sample bottle. Equal volumes of supernatant for each sample were mixed into quality control (QC) samples. The U3000 UPLC system coupled to a Q-Orbitrap mass spectrometer (Thermo Fisher Scientific, San Jose, CA, United States) with an electrospray (ESI) ionization source was used for the detection.

Import the resulting data into the Metaboanalyst 5.0 platform for analysis, partial least squares discriminant analysis (PLS-DA) and Orthogonal partial least squares analysis were used for the difference between groups discriminant analysis (OPLS-DA). The volcano map was drawn, and the serum differential metabolites were screened with Fold Change ≥ 1.2 or Fold Change ≤ 0.83 and *p* < 0.05. The pathway enrichment analysis was conducted using the Kyoto Encyclopedia of Genes and Genomes (KEGG), and the differential metabolic pathways were screened with *p* < 0.05 as the threshold value (Hong et al., 2023).

### 2.6 Statistical analysis

The statistical analysis of data was conducted with a *t*-test using GraphPad Prism 8.0.1. Weekly body weight was analyzed using GraphPad Prism 8.0.1 software’s two-way ANOVA. Results were expressed as means ± standard deviation (SD), and the differences were deemed significant at *p < 0.05, highly significant at **p < 0.01, and extremely significant at ***p < 0.001. For multiple comparisons across serum biomarkers, *p*-values were adjusted using the Holm–Bonferroni method to control the family-wise error rate, with significance set at *p* < 0.05 following the correction.

## 3 Results

### 3.1 Body weight

**Figure 1:**
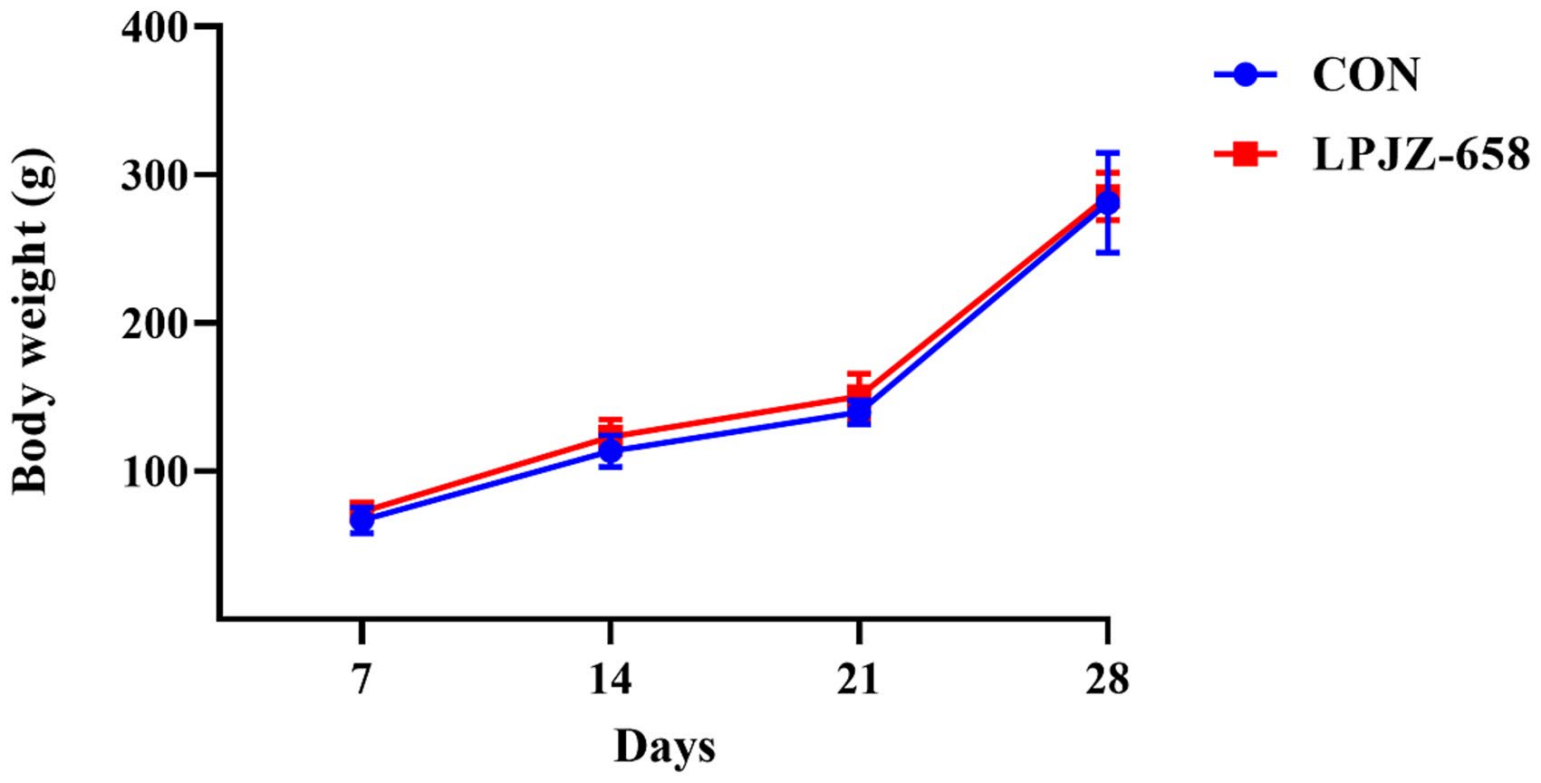
Effects of LPJZ-658 on weekly body weight of Luhua broiler.

The effect of supplementation of the basal diet with LPJZ-658 on the body weight of Luhua broiler is presented in Figure 1. The body weight of Luhua broilers in each group were recorded at the end of each week. The results showed supplementation with LPJZ-658 did not significantly alter the weekly body weight of Luhua broiler during days 1–28.

### 3.2 Immune function and antioxidant capacity

The effects of supplementation of LPJZ-658 for 28 days on the immune function and antioxidant capacity of Luhua broiler are shown in Table 1. Dietary supplementation with LPJZ-658 does not affect the spleen and thymus yield. Next, we examined the serum immunoglobulin levels of broiler in each group. The serum IgA was significantly higher in the LPJZ-658 group than in the CON group. Additionally, compared to the CON group, the activity of SOD in the LPJZ-658 group was significantly higher, while the concentration of MDA was markedly lower.

**Table 1:** Effects of LPJZ-658 on immune function and antioxidant capacity of Luhua broiler.

Items	CON	LPJZ-658	*p*-value
Thymus yield (%)	5.34 ± 3.40	3.29 ± 0.72	0.22
Spleen yield (%)	1.97 ± 0.79	2.33 ± 0.89	0.52
IgA (mg/mL)	0.99 ± 0.56	1.89 ± 0.71	0.02
IgG (mg/mL)	12.22 ± 8.67	16.70 ± 12.30	0.45
IgM (mg/mL)	13.99 ± 6.88	12.64 ± 3.03	0.64
SOD (U/mL)	14.90 ± 3.50	19.36 ± 0.91	0.0070
GSH-PX (U/mL)	247.86 ± 22.62	229.93 ± 16.77	0.13
MDA (nmol/mL)	16.55 ± 10.29	6.78 ± 3.95	0.04
T-AOC (mM)	0.95 ± 0.073	1.00 ± 0.70	0.28

### 3.3 Cecal microbiota analysis

**Figure 2:**
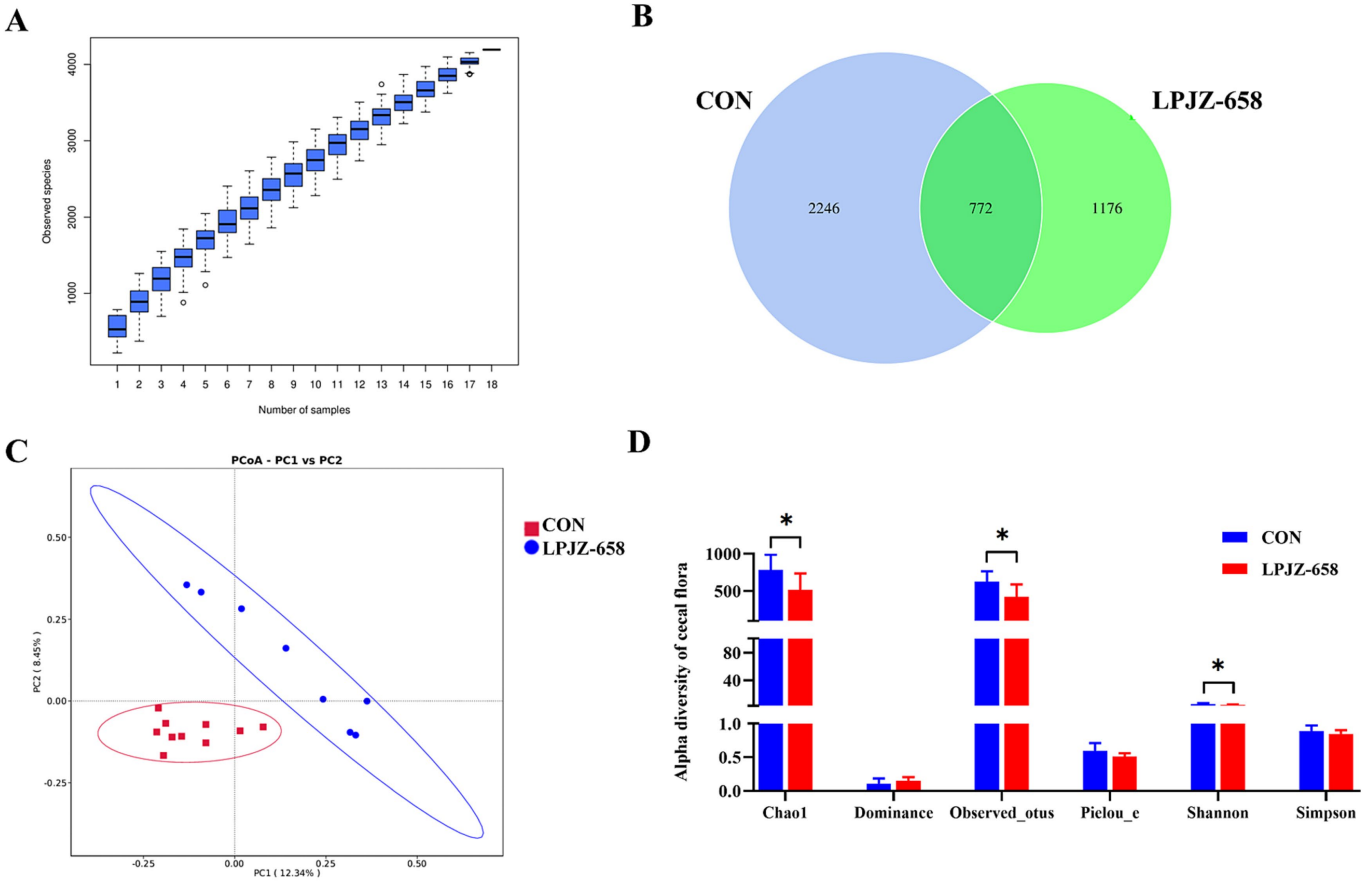
The effect of dietary supplementation with LPJZ-658 on the composition of cecal microbiota. **(A)** The species accumulation boxplot. **(B)** Venn diagram. **(C)** PCoA of cecal flora based on Jaccard distance. **(D)** Alpha diversity of cecal flora. Statistical significance was denoted by *p < 0.05, **p < 0.01 and ***p < 0.001.

The bacterial composition of cecal contents was profiled by 16S rRNA amplification analysis. In the species accumulation boxplot with the increase of the sample size of the two groups, the number of species did not increase, indicating that the sample size of the experiment was sufficient for further analysis (Figure 2A). As shown in Figure 2B, the Venn diagram generated after OTUs clustering with 97% homologous labels of all samples revealed that the CON and LPJZ-658 groups contained 3,018 and 1948 OTUs, respectively. The number of OTUs shared by the two groups amounted to 772, whereas the number of OTUs unique to the CON group was 2,246 OTUs and the number unique to the LPJZ-658 group was 1,176 OTUs. Notably, the genus Lactobacillus was distinctly identified in the unique OTUs of the LPJZ-658 group (data not shown). Following the supplementation of LPJZ-658 as an exogenous probiotic, specific colonization OTUs formed in the intestinal tract of Luhua broliers, manifested as unique Lactobacillus OTUs in this group.

To characterize the levels and patterns of diversity within individuals, different measures of alpha diversity were applied. As shown in Figure 2C, the Chao1, observed_otus, and Shannon index were significantly lower in the LPJZ-658 group compared with those in the CON group. The beta diversity of each group was calculated through PCoA (Principal Co-ordinates Analysis) based on the Jaccard distance algorithm. The PCoA diagram revealed that significant discrepancies existed between the microbial communities of each group (Figure 2D).

**Figure 3:**
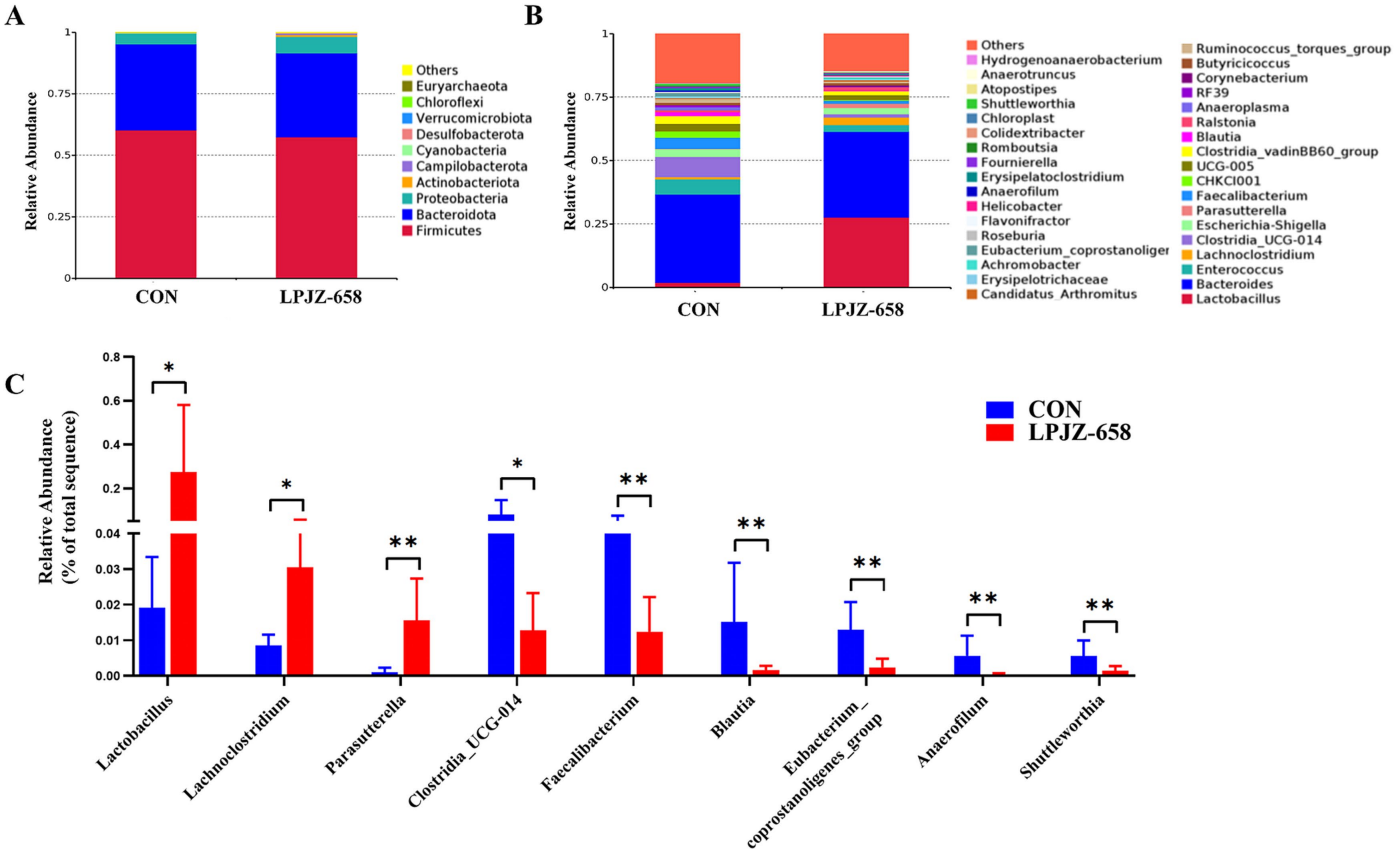
The effects of LPJZ-658 on composition of cecal flora of Luhua broiler. The relative abundance of microbial composition at phyla **(A)** and genus level **(B). (C)** Differential microbiota at the genus level. Statistical significance was denoted by *p < 0.05, **p < 0.01 and ***p < 0.001.

The microbial composition was further analyzed, and the Top 10 abundant phyla are shown in Figure 3A. Firmicutes, Bacteroidetes, and Proteobacteria were the dominant phyla in both CON and LPJZ-658 groups. At the genus level, the relative abundance of the TOP 35 species is shown in Figure 3B, and the microorganisms that differed at the genus level are shown in Figure 3C. The results showed that compared with the CON group, the relative abundance of Lactobacillus, Lachnoclostridium, and Parasutterella were significantly increased, and the relative abundance of Clostridia_UCG-014, Faecalibacterium, Blautia, Eubacterium_coprostanoligenes_group, Anaerofilum, and Shuttleworthia were markedly reduced.

**Figure 4:**
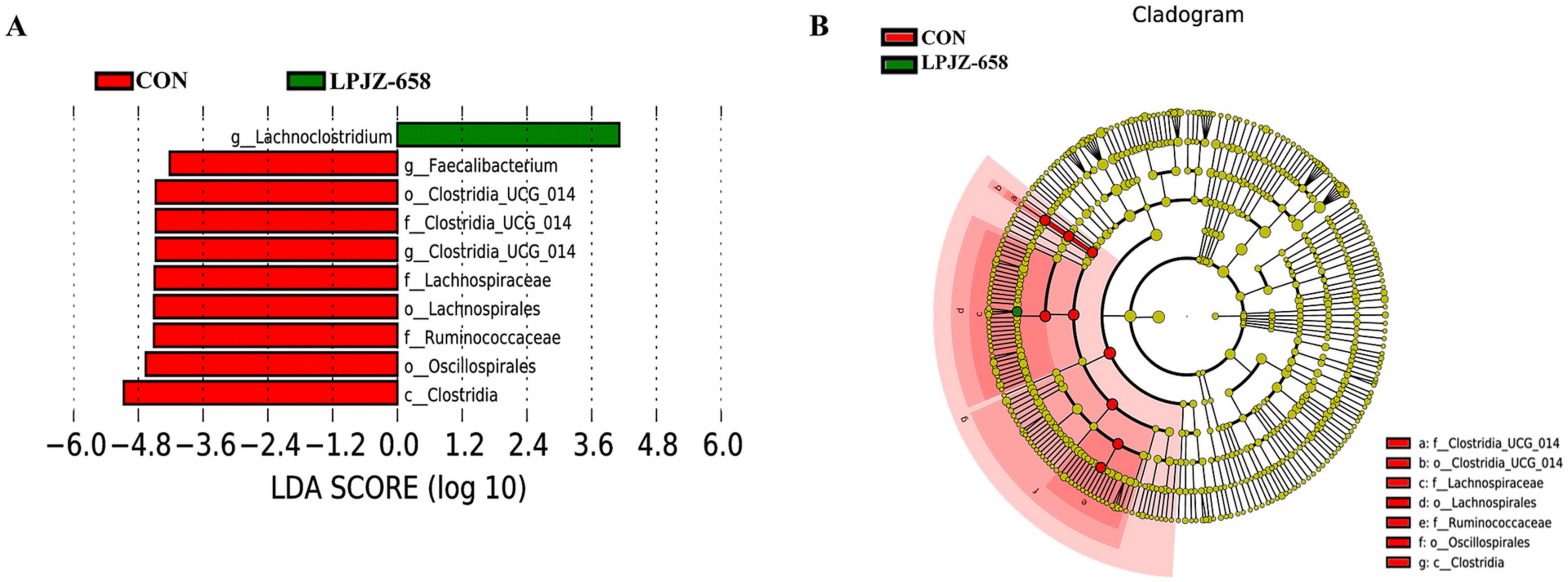
Linear discriminant analysis (LDA) effect size (LEfSe) analysis of cecal microbiota. **(A)** Distribution histogram of linear discriminant analysis (LDA) values (LDA score = 4). **(B)** Evolutionary branching diagram.

Linear discriminant analysis (LDA) effect size (LefSe) analysis was also performed to confirm the different effects of LPJZ-658 on cecal microbiota in Luhua broiler (Figures 4A,B), which was used to identify biomarkers with statistical differences between the two groups based on all levels. As shown in Supplementary Figure S1, Compared with the CON group, Lachnoclostridium at the genus level was significantly increased, and Clostridia_UCG_014 and Faecalibacterium at the genus level were significantly decreased in the LPZJ-658 group. Clostridia at the class level and Lachnospirales, Oscillospirales, and Clostridia_UCG_014 at the order level in the LPJZ-658 group were significantly decreased compared with those in the CON group. In addition, Ruminococcaceae, Lachnospiraceae, and Clostridia_UCG_014 at the family level were also significantly decreased in the LPJZ-658 group compared with the CON group.

**Figure 5:**
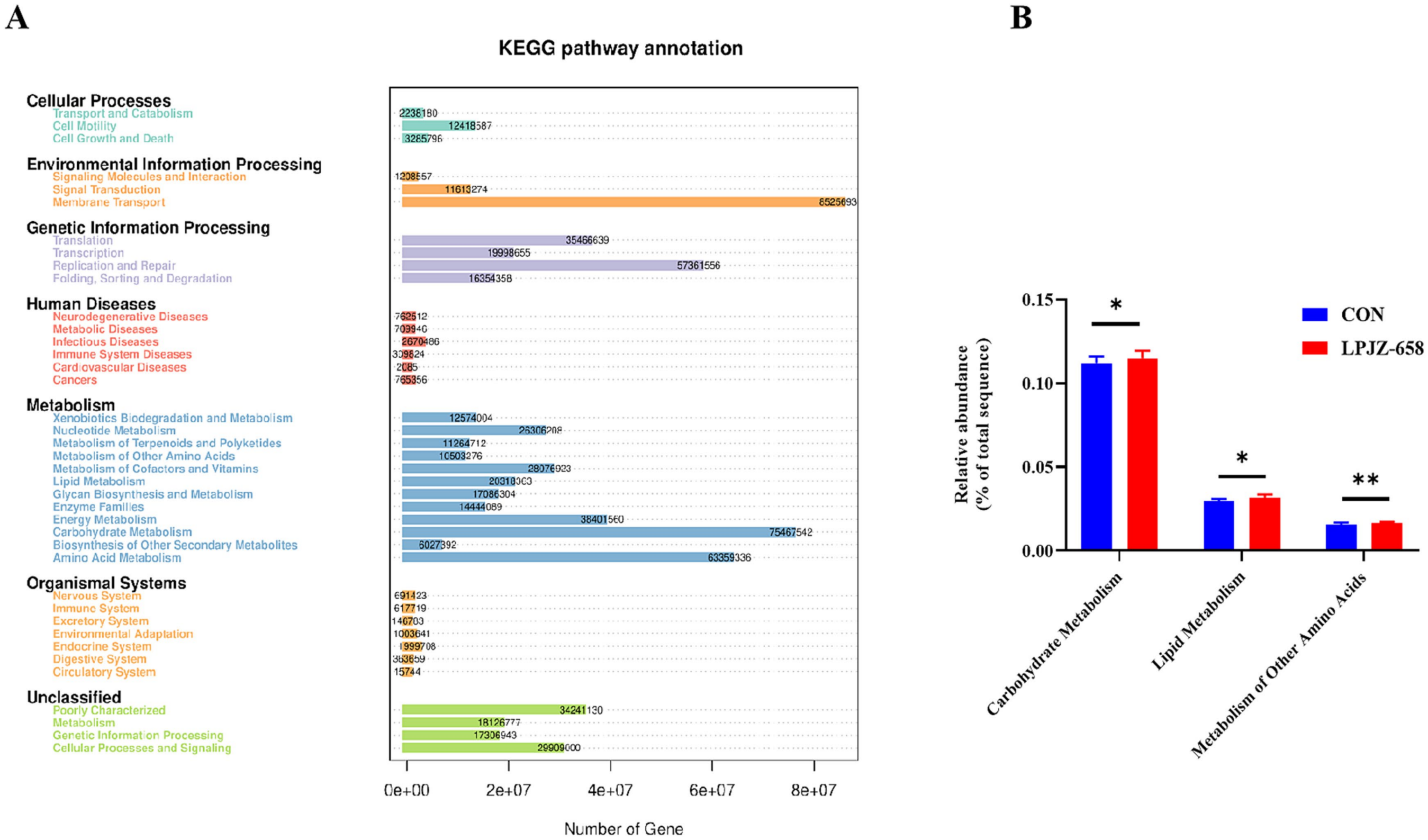
PICRUSt predicted analysis **(A)** and significantly changed metabolic pathways **(B)**.

We then used the PICRUSt (Phylogenetic Investigation of Communities by Reconstruction of Unobserved States) analysis to predict the microbial metabolic function of the two groups. The predicted results showed that the metabolic functions of the microorganisms in LPJZ-658 mainly include membrane transport, carbohydrate metabolism, and amino acid metabolism (Figure 5A). Furthermore, the predictive values of Carbohydrate Metabolism, Lipid Metabolism, and Metabolism of Other Amino Acids in the LPJZ-658 group were significantly higher than those in the CON group (Figure 5B).

### 3.4 Serum metabolome profile and biomarker annotation

**Figure 6:**
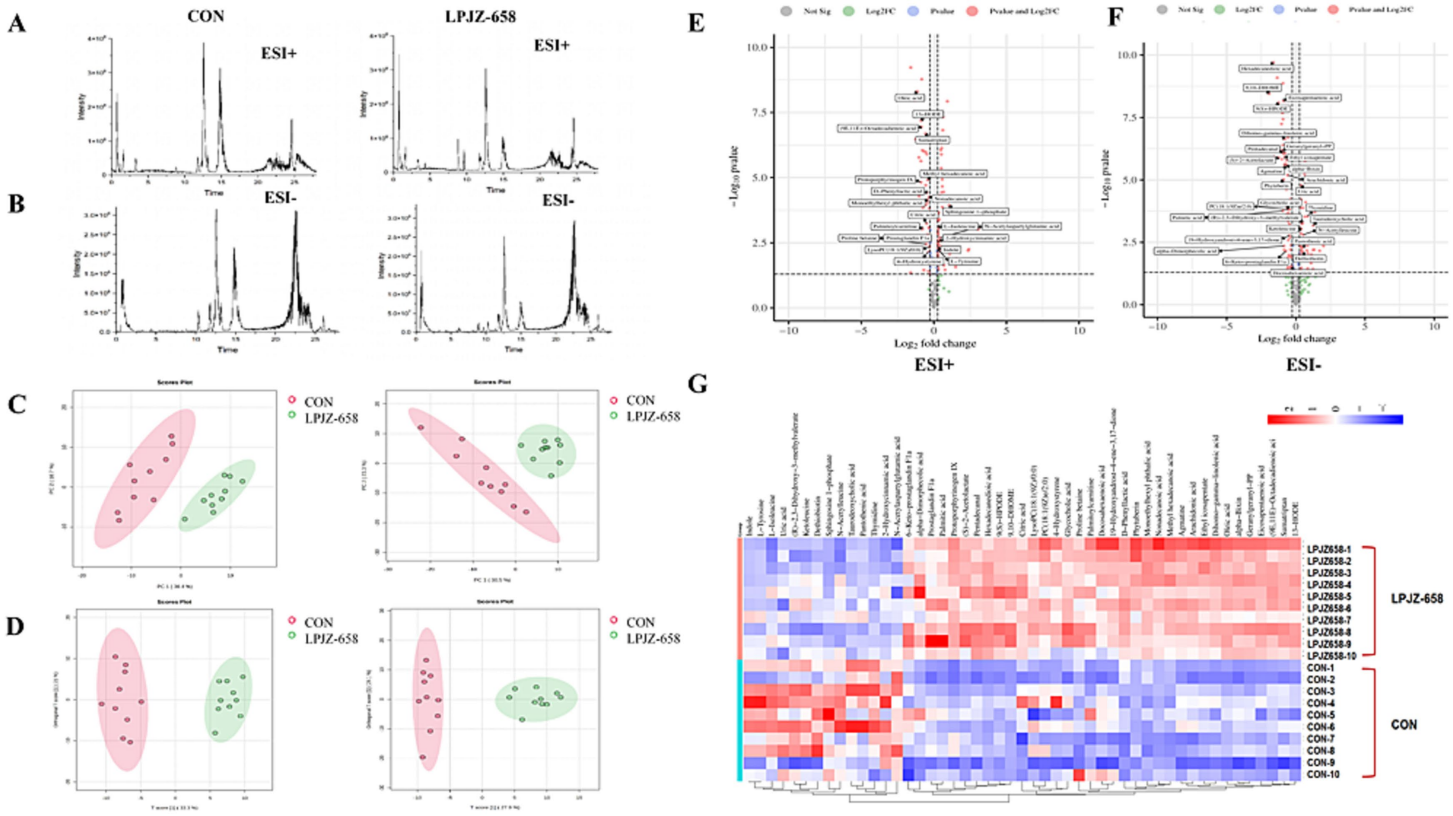
Changes in the metabolite profiles between the two groups. Representative base peak chromatograms of serum samples acquired from 50% methanol in water extracts [**(A)** ESI+ and **(B)** ESI−]. **(C)** PCA score plots of the CON and LPJZ-658 group, based on the data acquired from 50% methanol in water extracts (left, ESI+, and right, ESI−). **(D)** OPLS-DA score plots of CON and LPJZ-658 group based on the data acquired from 50% methanol in water extracts (left, ESI+, and right, ESI−). The volcano diagram [**(E)** ESI+ and **(F)** ESI−]. **(G)** Heatmap of changes in the intensity of biomarkers in two groups.

We then performed non-targeted metabolomics by using UHPLC-Q-Orbitrap/MS to understand changes in serum metabolites between the two groups. By selecting the base peak where the characteristic fragments of metabolites are located, it was evident that these peaks differed significantly in terms of peak intensity and retention time (Figures 6A,B). Following the generation of the Progenesis QI data, datasets, including the sample information, RT m/z values, and peak intensities, were generated for statistical analysis. Principal component analysis (PCA) was drawn according to the LC–MS data of serum extract in two modes of positive and negative ions, as shown in Figure 6C. The CON group and LPJZ-658 group were separated, and there were obvious metabolic changes between the two groups. The dispersion point of OPLS-DA is shown in Figure 6D. VIP > 1 characteristic marker was selected to screen the differential metabolites, to determine the serum metabolites that contributed the most to the difference between the two groups of samples. Furthermore, specific serum metabolites were screened with Fold Change > 1.2 or Fold Change < 0.83 and *p* < 0.05 as the standard. A total of 49 differential metabolites were identified between the CON and LPJZ-658 groups (Supplementary Table S2) and shown in the volcano diagram (Figures 6E,F). Finally, we compared the differences in metabolic compounds in all serum samples of the two groups, and these differences in metabolites were shown in the heat map (Figure 6G).

### 3.5 Metabolic pathway analysis

**Figure 7:**
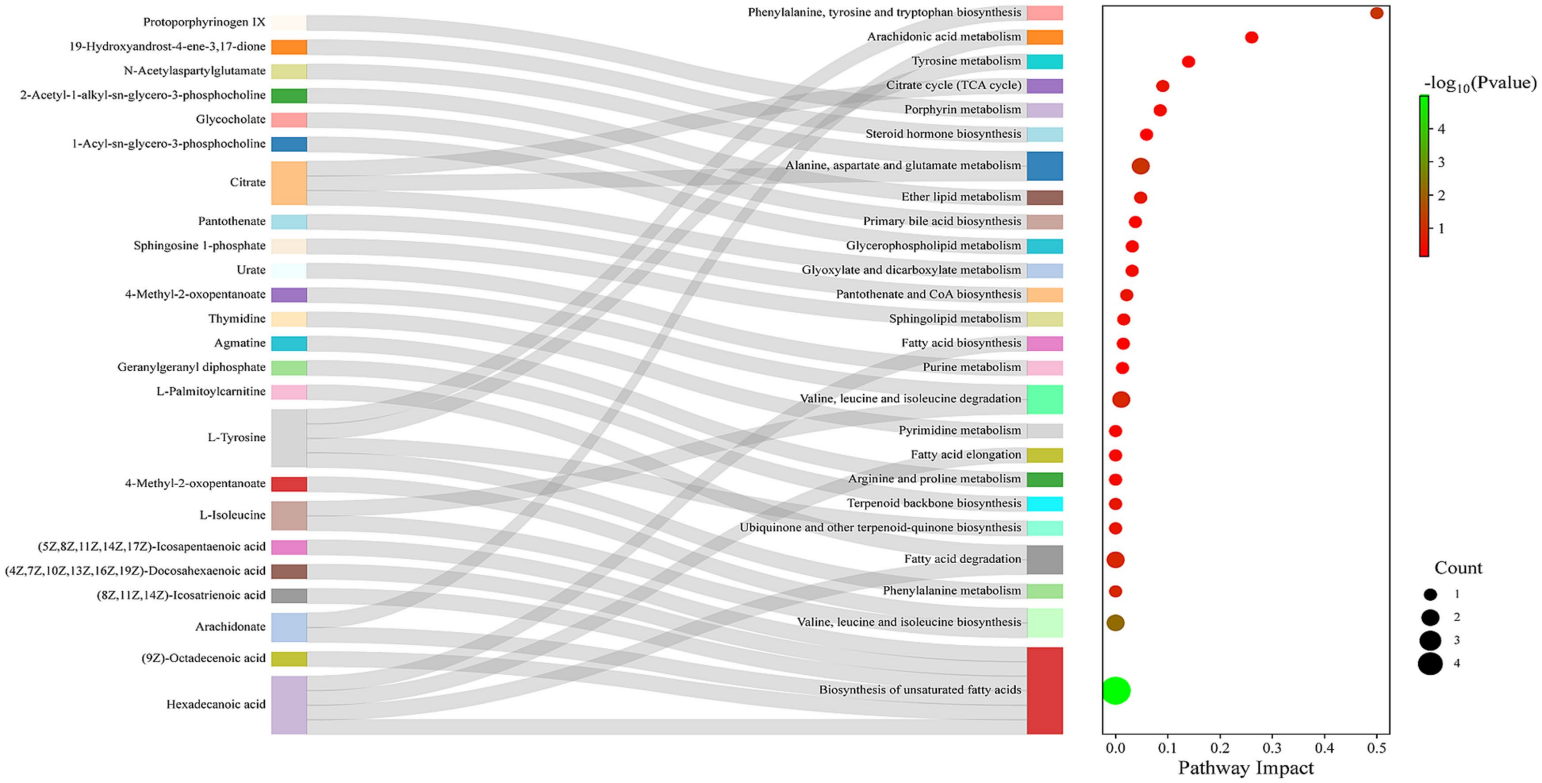
Sankey diagram of metabolic pathway analysis.

According to the Sankey diagram (Figure 7), these differential metabolites were mainly related to phenylalanine, tyrosine and tryptophan biosynthesis, arachidonic acid metabolism, tyrosine metabolism, citrate cycle, and Alanine, aspartate and glutamate metabolism. The key differential metabolites involved mainly include *N*-Acetylaspartylglutamate, Citric acid, Arachidonic acid, and L-Tyrosine.

### 3.6 Correlations between serum differential metabolites and caecum bacteria

**Figure 8:**
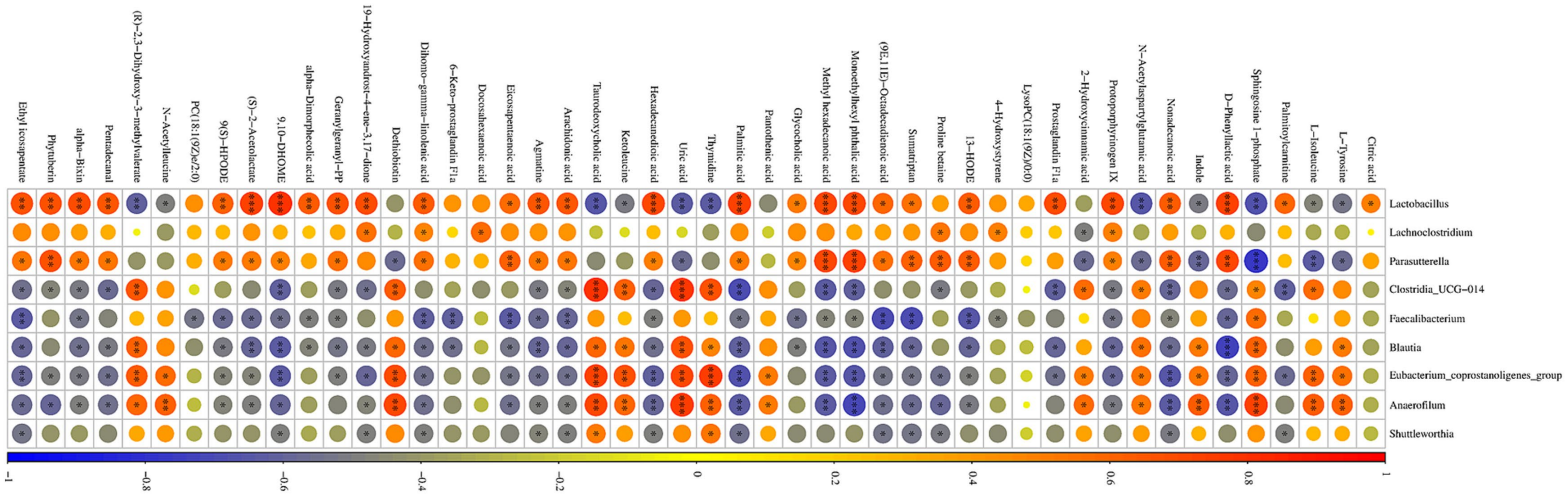
Correlation analysis among differential serum metabolites and caecum microbiota.

Correlations between two groups of differential caecum bacteria at the genus level and differential serum metabolites were elucidated by Spearman correlation analysis and visualization network (Figure 8). The analysis showed that the increased bacteria due to supplementation of LPJZ-658 showed a consistent correlation with all metabolites. For example, Lactobacillus, Lachnoclostridium, Parasutterella with Citric acid, Palmitoylcarnitine, D-Phenyllactic acid, and Nonadecanoic acid. Protoporphyrinogen IX, Prostaglandin F1a, 13-HODE, Sumatriptan, (9E,11E)-Octadecadienoic acid, Monoethylhexyl phthalic acid, Methyl hexadecanoic acid, Glycocholic acid, and other metabolites showed positive correlations or a trend, while the decreased bacteria (Clostridia_UCG-014, Faecalibacterium, Blautia, Eubacterium_coprostanoligenes_group, Anaerofilum, and Shuttleworthia) showed negative correlations or a trend with these metabolites. It is indicated that the regulatory effects of LPJZ-658 on serum metabolites and metabolic pathways might be associated with the abundance of different intestinal microbiota.

## 4 Discussion

Nutritional supplementation of poultry at a young age has become one of the most important factors affecting poultry production (Song B. et al., 2022; Liu M. et al., 2023). The primary objective of this study was to investigate the effect of dietary supplementation of LPJZ-658 on the growth performance and gut microbiota of Luhua broiler from early life. Yang et al. (2024) showed that during the starter period (1–21 days), the broiler fed with *L. plantarum* HJLP-1 had a higher average weight daily gain. Notably, our previous study has shown that feeding LPJZ-658 in the early growth stage of broiler can significantly increase the ileum and cecum length, duodenum and ileum villus height, and ileum villus height/crypt depth ratio, which may be one of the reasons for increasing the growth performance of broiler (Liu et al., 2023b). To our surprise, in this experiment, supplementation of LPJZ-658 for 28 days had no significant effect on the body weight of Luhua broiler. However, probiotics also play a role by enhancing host immunity, intestinal microbiome and metabolome (Jäger et al., 2018; Ma et al., 2023), which led us to conduct further studies. A higher level of immunity can help chicks effectively resist the invasion of various pathogens and maintain a healthy state. Establishing a healthy immune level in the early stage can stimulate the development and maturity of the chick’s immune system, laying a good immune foundation for its subsequent growth stage. Serum immunoglobulin plays an important role in the regulation of immune function and is a key indicator of humoral immunity (Zhao et al., 2023; Yang et al., 2024). It has been shown that dietary supplementation with *L. plantarum* significantly increased the immunoglobulin content of chicks (Song X. et al., 2022). In this experiment, LPJZ-658 significantly elevated the serum IgA level of Luhua broiler. Metabolic activities constantly generate free radicals, which when in excess cause oxidative stress, severely damaging all biomolecules, thus affecting organismal homeostasis and even leading to disease and death (Amir Aslani and Ghobadi, 2016). Some animal experiments have shown that probiotics can stimulate the host’s antioxidant system and increase the level of serum antioxidant enzymes (Liao et al., 2015; Qin et al., 2024). Broiler fed with probiotics decreased malondialdehyde levels in jejunal mucosa and serum, and the increased activities of hepatic GSH-PX and jejunal CAT were observed (Wu et al., 2019). Similar to these studies, dietary supplementation of LPJZ-658 significantly increased serum SOD activity and significantly decreased serum MDA concentration in Luhua broiler.

Although LPJZ-658 did not affect the body weight of Luhua broiler in this study. Notably, a previous study dedicated that increased Lactobacillus in the gut microbiota of newborn broiler chicks and ducks could result in weight gain increase, but the differences in the intestinal microbiota may precede weight increase (Angelakis and Raoult, 2010). The cecum of poultry contains a large number of microorganisms, and the composition of the microbiome will change with age and maintain and regulate the intestinal microecological balance through its metabolism (Robles Alonso and Guarner, 2013; Wang et al., 2023). The balance and stability of the gut microbiota play an important role in maintaining the growth and development of the body, nutrient digestion and absorption, and immune antagonism. Numerous studies have shown that *L. plantarum* is beneficial to the intestinal homeostasis of poultry and improves performance by regulating the populations of beneficial bacteria and potential pathogens in the gut (An et al., 2022; Yin et al., 2023). The results of 16S rRNA high-throughput sequencing showed significant differences in Beta diversity between the two groups, confirming that LPJZ-658 could change the cecal microflora composition of Luhua broiler.

By analyzing the bacterial flora of the cecum contents, we found that the main bacteria in the cecum flora of Luhua broiler included Firmicutes, Bacteroidetes, and Proteobacteria, which was consistent with the results of other studies (Vimon et al., 2023). Compared with the CON group in the genus level, Luhua broiler fed LPJZ-658 showed high expression of Lactobacillus in the genus caecum, which can reduce gut pH by metabolizing high concentrations of lactic acid, which inhibits the growth of other bacteria (Shokryazdan et al., 2017). In addition, other bacteria genera with significant increases in the LPJZ-658 group included Lachnoclostridium and Parasutterella genera. As the common members of the intestine, Lachnoclostridium forms an important part of the gut microbiome, which can play an anti-inflammatory role and maintain intestinal health through its metabolites, especially butyrate (Chen et al., 2021). Studies have confirmed significant changes in aromatic amino acids, bilirubin, purines, and bile acid derivatives in the metabolites of Parasutterella, and changes in bile acid profiles were consistent with altered expression of ileal bile acid transporter protein genes and hepatic bile acid synthesis genes, suggesting a potential role for Parasutterella in bile acid maintenance and cholesterol metabolism (Ju et al., 2019). In this study, we observed an increase in the number of these three genera in the cecal contents of Luhua broiler fed LPJZ-658, which is in general agreement with the results reported in a recent study (Qin et al., 2024). These results indicated that supplementation of LPJZ-658 could increase the abundance of beneficial bacteria in the intestinal tract of Luhua broiler in the early growth stage. Of the six bacteria genera in which LPJZ-658 was significantly down-regulated, Clostridia_UCG-014 was a key bacterium closely associated with the development of inflammation (Hu et al., 2022). Faecalibacterium also has been linked to inflammation, especially in inflammatory bowel disease (IBD). The relative presence of this genus is thought to partly reflect intestinal health (Martín et al., 2023). Blautia is considered to be a biomarker of metabolic disease risk (Benítez-Páez et al., 2020). This suggests that LPJZ-658 may have a probiotic effect by regulating the microbiota closely associated with inflammation. However, the abundance expression of Eubacterium_coprostanoligenes_group was reduced, which has been proven to produce beneficial short-chain fatty acids with anti-inflammatory effects (Wei et al., 2021). This evidence suggests that LPJZ-658 may also reduce the abundance of some beneficial bacteria. In addition, we also observed lower levels of Anaerofilum and Shuttleworthia in the LPJZ-658 group. They are thought to be associated with metabolic or chronic diseases (Guldris et al., 2017; Lee et al., 2020). Changes in metabolites within the serum of early-growth Luhua broiler were examined by non-targeted metabolomics with LPJZ-658 intervention. Significant segregation between the two groups was observed in ESI+ and ESI− in PCA and OPLS-DA analysis, suggesting that supplementation with LPJZ-658 had a significant impact on metabolism in early-growth Luhua broiler. LPJZ-658 upregulated 35 metabolites, such as alpha-Dimorphecolic acid, Prostaglandin Fla., Palmitic acid, Protoporphyrinogen IX, (S)-2-Acetolactate, Citric acid, and Arachidonic acid, and downregulated 14 metabolites, including Indole, L-Tyrosine, M-Lsoleucine, and Uric acid.

Pathway enrichment prediction analysis revealed that LPJZ-658 is involved in regulating metabolic pathways including phenylalanine, tyrosine and tryptophan biosynthesis, arachidonic acid metabolism, tyrosine metabolism, TCA cycle, alanine, aspartate and glutamate metabolism. The main serum differential metabolites are *N*-Acetylaspartylglutamic acid, citric acid, arachidonic acid, and L-Tyrosine. This was generally consistent with the results of microbial metabolism PICRUSt in the LPJZ-658 group, where metabolism of other amino acids, lipid metabolism, and carbohydrate metabolism were significantly higher than those in the control group. Citric acid plays a vital role in the body’s metabolism, and the TCA cycle serves as the ultimate common oxidation pathway for carbohydrates, fats, and amino acids (Akram, 2014). It is essential for aerobic glycolysis and supplies ATP, lipids, and non-essential amino acids for rapid cell proliferation (Zhu and Thompson, 2019). Arachidonic acid (AA) is a key mediator in inflammatory responses and plays an important role in immune reactions. Arachidonic acid can be further metabolized into prostaglandin G2 (PGG2) and prostaglandin H2 (PGH2) (Uribe et al., 2018). Whereas L-Tyrosine is an important nutritionally essential amino acid that plays an important role in metabolism, growth, and development in humans and animals (Wypych et al., 2021). *N*-acetylaspartylglutamic acid (NAAG) is an endogenous neuropeptide that plays a significant role in the central nervous system (CNS) (Neale, 2011). Spearman correlation analysis of differential microbial groups and differential metabolites showed that Lactobacillus, Lachnoclostridium, and Parasutterella, whose abundance increased due to supplementation with LPJZ-658, had a positive correlation or a trend toward positive correlation with citric acid and arachidonic acid, and a negative correlation or a trend toward negative correlation with *N*-acetylaspartylglutamic acid and L-tyrosine. Hu et al. observed that in the hosts treated with *Akkermansia muciniphila*, the levels of key metabolites in the TCA cycle, such as citric acid and *α*-ketoglutarate, were significantly increased (Hu et al., 2025). Moreover, by restoring the transcriptional levels of key enzymes in the TCA cycle, including succinate dehydrogenase (Sdha, Sdhb, Sdhc), isocitrate dehydrogenase (Idh3a, Idh2), and pyruvate dehydrogenase (Pdhb), it was confirmed that exogenous probiotics might support metabolic and regenerative processes by enhancing the efficiency of the TCA cycle (Hu et al., 2025). Our research results indicated that the upregulation of citric acid levels and enrichment of the TCA cycle after LPJZ-658 treatment may be achieved by regulating the transcriptional levels of key enzymes. Uribe’s indicated oral gavage of healthy host with LbGG for 5 days leads to increased levels of AA and COX-2 protein expression in the colonic mucosa (Uribe et al., 2018). In our study, LPJZ-658 increased the level of arachidonic acid in serum, and prostaglandin F1α (PGF1α), another metabolite of AA, was also significantly enriched upon exposure to LPJZ-658 (Figure 6G). In our research report, the impact of supplemental Lactobacillus on the amino acid metabolism of Luhua broilers was significant, particularly evidenced by the consumption of L-tyrosine and *N*-acetylaspartylglutamic acid. The microbiota can influence the ThiH (2-iminoglycine synthase)-mediated metabolism of L-tyrosine into p-cresol sulfate (PCS) (Wypych et al., 2021), which may be one of the main reasons for the negative correlation observed between the enriched bacterial genera and L-tyrosine. Glutamic acid and aspartic acid are precursors to *N*-acetylaspartylglutamic acid, and Lactobacillus may utilize them as a carbon source, thereby reducing the substrate supply available for the host’s *N*-acetylaspartylglutamic acid synthesis. Another potential cause could be the inhibition of glutaminase or *N*-acetylaspartylglutamic acid synthase activity, which would consequently lower *N*-acetylaspartylglutamic acid levels. However, these hypotheses require confirmation through further studies. Conversely, the correlation of metabolites with Clostridia_UCG-014, Faecalibacterium, Blautia, Eubacterium_coprostanoligenes_group, Anaerofilum, and Shuttleworthia, whose abundance decreased due to supplementation with LPJZ-658, was exactly the opposite of what was described above. This reflects that early supplementation with LPJZ-658 in Luhua broiler may achieve regulatory effects on phenylalanine, tyrosine and tryptophan biosynthesis, arachidonic acid metabolism, tyrosine metabolism, and citrate cycle, by the regulation of the caecum microbial environment. Our investigation demonstrated that the supplementation of LPJZ-658 for 28 days significantly increased the serum IgA level and antioxidant capacity of Luhua broiler. Meanwhile, our study revealed that LPJZ-658-alterated caecum microbiota significantly correlated with serum metabolome, suggesting a potential mechanism of LPJZ-658 in altering phenylalanine, tyrosine and tryptophan biosynthesis, arachidonic acid metabolism, and tyrosine metabolism by modulating the caecum microbiota.

## Data Availability

The data presented in the study are deposited in the Sequence Read Archive (SRA) repository, accession number PRJNA1302116.
