# Supplementary material for: Effect of Lactiplantibacillus plantarum LPJZ-658 on caecum microbiota and serum metabolomics of Luhua broiler
Source: Front Microbiol. 2025 Aug 20;16:1622009. doi: 10.3389/fmicb.2025.1622009 (PMC12405372; doi:10.3389/fmicb.2025.1622009)
Supplement: Supplementary file 1 [file Supplementary_file_1.docx]

Supplementary Material

# Supplementary Figures and Tables


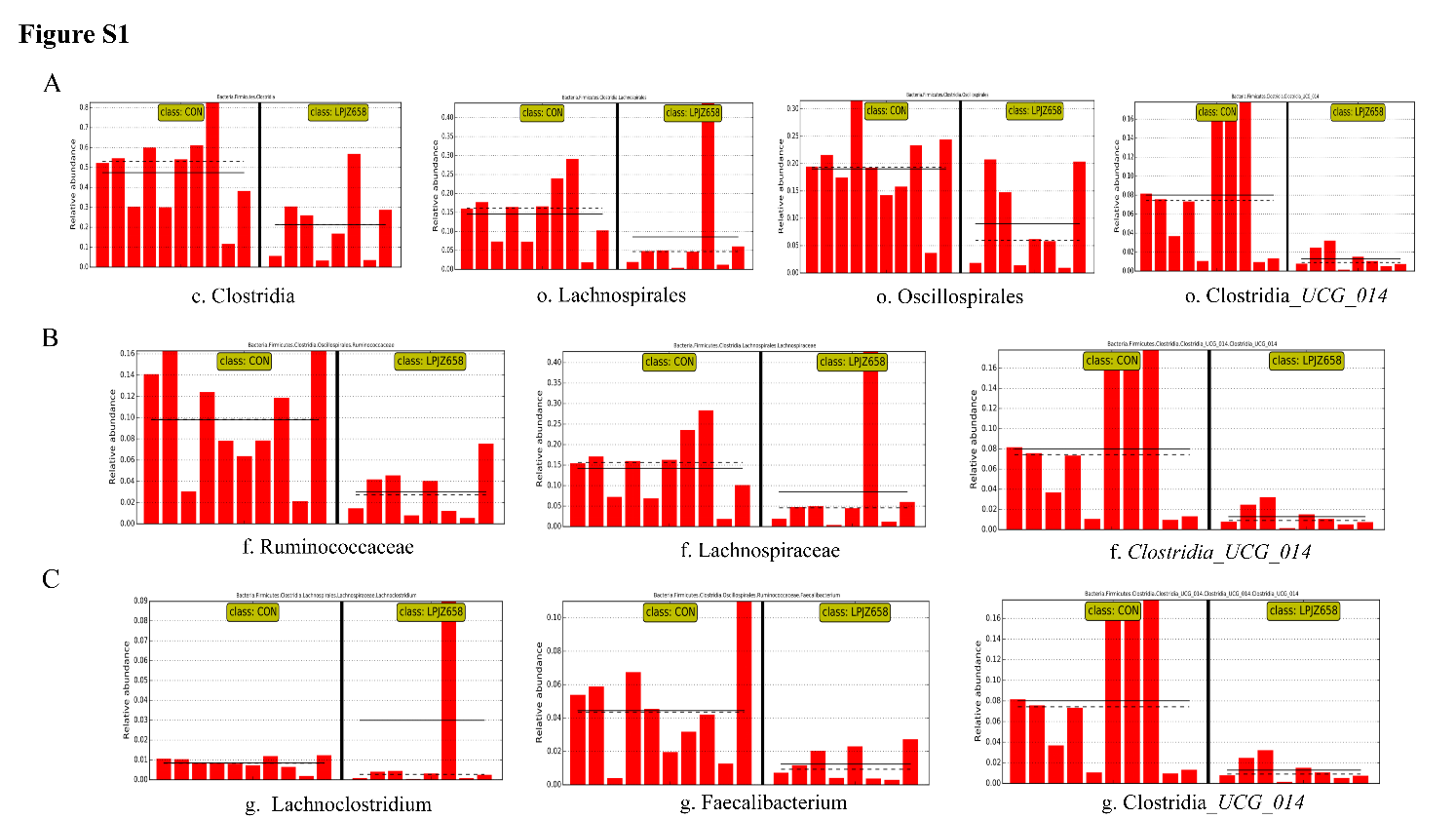


**Supplementary Figure 1.** Biomarkers in LEfSe analysis. (A) Class level and order level; (B) Family level; (C) Genus level.

**Table S1. Composition and nutrient levels of the experimental basal diet (%).**

| **Ages** | **1-21d** | **22d-28d** |
| --- | --- | --- |
| Ingredients, % |  |  |
| Corn | 58.80 | 65.30 |
| Bean pulp | 35.60 | 30.00 |
| Soybean oil | 1.30 | 0.50 |
| Premix^1^ | 0.34 | 0.34 |
| Dicalcium phosphate | 1.40 | 1.30 |
| Salt | 0.26 | 0.26 |
| Limestone | 1.50 | 1.62 |
| Sodium bicarbonate | 0.20 | 0.20 |
| DL-methionine (98.5%) | 0.30 | 0.25 |
| L-lysine sulfate (78%) | 0.20 | 0.13 |
| Choline chloride (60%) | 0.10 | 0.10 |
| Nutritional level^2^ |  |  |
| Metabolic energy, MJ/kg | 12.14 | 12.14 |
| Crude protein, % | 20.00 | 18.00 |
| Coarse fibre, % | 2.77 | 2.58 |
| Crude fat, % | 3.62 | 2.99 |
| Methionine, % | 0.59 | 0.52 |
| Methionine+cystine, % | 0.91 | 0.82 |
| Lysine, % | 1.17 | 1.00 |
| Ca, % | 1.00 | 1.00 |
| Total phosphorus, % | 0.60 | 0.56 |
| Acailable phosphorus, % | 0.41 | 0.38 |

Content by kg of =diets^1^Premix:Vit.D3=400IU; Vit.A=1000IU; Vit.E=20IU; Vit.B=120.02mg; Vit.K=15.5mg; Folic acid=0.55mg; Choline=2500mg; Vit.B1=2.5mg; Pantothenic acid=15mg; Pyridoxine=5mg; Riboflavin=6.5mg; Fe=80mg; Zn=40mg; Cu=8mg; Mn=60mg; Se=0.15mg; I=0.35mg; Phytase=2000FTU.

^2^All nutritional components are calculated values.

**Table S2. The information of the annotated metabolite.**

| No. | Annotation | RT (min) | | Detected m/z | Formular | Adducts |
| --- | --- | --- | --- | --- | --- | --- |
| 1 | Citric acid | | 0.87 | 215.0161 | C_6_H_8_O_7_ | [M+Na]+ |
| 2 | Glycocholic acid | | 11.30 | 500.2794 | C_26_H_43_NO_6_ | [M+Cl]- |
| 3 | L-Tyrosine | | 3.34 | 146.0601 | C_9_H_11_NO_3_ | [M+H-2H_2_O]+ |
| 4 | L-Isoleucine | | 1.09 | 132.1020 | C_6_H_13_NO_2_ | [M+H]+ |
| 5 | Oleic acid | | 16.77 | 265.2524 | C_18_H_34_O_2_ | [M+H-H_2_O]+ |
| 6 | Pantothenic acid | | 2.24 | 218.1032 | C_9_H_17_NO_5_ | [M-H]- |
| 7 | Palmitic acid | | 16.37 | 255.2333 | C_16_H_32_O_2_ | [M-H]- |
| 8 | Palmitoylcarnitine | | 10.73 | 400.3417 | C_23_H_45_NO_4_ | [M+H]+ |
| 9 | Thymidine | | 1.74 | 241.0831 | C_10_H_14_N_2_O_5_ | [M-H]- |
| 10 | Sphingosine 1-phosphate | | 10.66 | 380.2556 | C_18_H_38_NO_5_P | [M+H-H_2_O]+ |
| 11 | Uric acid | | 0.87 | 167.0203 | C_5_H_4_N_4_O_3_ | [M-H]- |
| 12 | D-Phenyllactic acid | | 10.22 | 167.0702 | C_9_H_10_O_3_ | [M+H-H_2_O]+ |
| 13 | Hexadecanedioic acid | | 10.89 | 285.2076 | C_16_H_30_O_4_ | [M-H]- |
| 14 | Ketoleucine | | 4.16 | 129.0546 | C_6_H_10_O_3_ | [M-H]- |
| 15 | Indole | | 3.34 | 118.0653 | C_8_H_7_N | [M+H]+ |
| 16 | Nonadecanoic acid | | 9.68 | 316.3206 | C_19_H_38_O_2_ | [M+NH_4_]+ |
| 17 | Taurodeoxycholic acid | | 10.33 | 498.2905 | C_26_H_45_NO_6_S | [M-H]- |
| 18 | Arachidonic acid | | 14.71 | 303.2334 | C_20_H_32_O_2_ | [M-H]- |
| 19 | N-Acetylaspartylglutamic acid | | 23.81 | 327.0777 | C_11_H_16_N_2_O_8_ | [M+Na]+ |
| 20 | Protoporphyrinogen IX | | 7.96 | 1159.5890 | C_34_H_40_N_4_O_4_ | [2M+Na]+ |
| 21 | Agmatine | | 14.71 | 305.2399 | C_5_H_14_N_4_ | [2M+FA-H]- |
| 22 | Eicosapentaenoic acid | | 13.47 | 301.2178 | C_20_H_30_O_2_ | [M-H]- |
| 23 | Docosahexaenoic acid | | 14.33 | 327.2336 | C_22_H_32_O_2_ | [M-H]- |
| 24 | 2-Hydroxycinnamic acid | | 0.87 | 182.0812 | C_9_H_8_O_3_ | [M+H]+ |
| 25 | Prostaglandin F1a | | 16.35 | 395.2199 | C_20_H_36_O_5_ | [M+K]+ |
| 26 | LysoPC(18:1(9Z)/0:0) | | 12.90 | 522.3550 | C_26_H_52_NO_7_P | [M+H]+ |
| 27 | 6-Keto-prostaglandin F1a | | 8.55 | 369.2289 | C_20_H_34_O_6_ | [M-H_2_O-H]- |
| 28 | Dihomo-gamma-linolenic acid | | 15.75 | 305.2491 | C_20_H_34_O_2_ | [M-H]- |
| 29 | Dethiobiotin | | 4.14 | 259.1304 | C_10_H_18_N_2_O_3_ | [M+FA-H]- |
| 30 | 19-Hydroxyandrost-4-ene-3,17-dione | | 14.33 | 301.1813 | C_19_H_26_O_3_ | [M-H]- |
| 31 | 4-Hydroxystyrene | | 11.86 | 184.0731 | C_8_H_8_O | [M+ACN+Na]+ |
| 32 | Geranylgeranyl-PP | | 16.80 | 431.1753 | C_20_H_36_O_7_P_2_ | [M-H_2_O-H]- |
| 33 | 13-HODE | | 13.64 | 279.2316 | C_18_H_32_O_3_ | [M+H-H_2_O]+ |
| 34 | alpha-Dimorphecolic acid | | 11.46 | 295.2282 | C_18_H_32_O_3_ | [M-H]- |
| 35 | 9,10-DHOME | | 11.98 | 313.2390 | C_18_H_34_O_4_ | [M-H]- |
| 36 | Proline betaine | | 0.80 | 144.1019 | C_7_H_13_NO_2_ | [M+H]+ |
| 37 | Sumatriptan | | 15.00 | 337.1667 | C_14_H_21_N_3_O_2_S | [M+ACN+H]+ |
| 38 | (9E,11E)-Octadecadienoic acid | | 15.00 | 263.2367 | C_18_H_32_O_2_ | [M+H-H_2_O]+ |
| 39 | (S)-2-Acetolactate | | 2.06 | 131.0339 | C_5_H_8_O_4_ | [M-H]- |
| 40 | 9(S)-HPODE | | 11.38 | 311.2233 | C_18_H_32_O_4_ | [M-H]- |
| 41 | PC(18:1(9Z)e/2:0) | | 15.35 | 594.3789 | C_28_H_56_NO_7_P | [M+FA-H]- |
| 42 | N-Acetylleucine | | 1.36 | 172.0972 | C_8_H_15_NO_3_ | [M-H]- |
| 43 | (R)-2,3-Dihydroxy-3-methylvalerate | | 4.64 | 129.0546 | C_6_H_12_O_4_ | [M-H_2_O-H]- |
| 44 | Monoethylhexyl phthalic acid | | 12.02 | 301.1407 | C_16_H_22_O_4_ | [M+Na]+ |
| 45 | Pentadecanal | | 11.30 | 271.2284 | C_15_H_30_O | [M+FA-H]- |
| 46 | alpha-Bixin | | 16.80 | 429.1801 | C_25_H_30_O_4_ | [M+Cl]- |
| 47 | Phytuberin | | 9.57 | 293.1763 | C_17_H_26_O_4_ | [M-H]- |
| 48 | Ethyl icosapentate | | 15.09 | 329.2492 | C_22_H_34_O_2_ | [M-H]- |
| 49 | Methyl hexadecanoic acid | | 8.87 | 288.2894 | C_17_H_34_O_2_ | [M+NH_4_]+ |
